# Constitutive TDO2 expression promotes liver cancer progression by an autocrine IL-6 signaling pathway

**DOI:** 10.1186/s12935-021-02228-9

**Published:** 2021-10-17

**Authors:** Zhengzhong Wu, Leye Yan, Junqing Lin, Kun Ke, Weizhu Yang

**Affiliations:** Department of Interventional Radiology, Fujian Medical University Union Hospital, No 29, Xinquan Road, Fuzhou, 350001 China

**Keywords:** Tryptophan metabolism, TDO2, AhR, IL-6, Liver cancer

## Abstract

**Background:**

Increased tryptophan (Trp) metabolism by indoleamine 2,3-dioxygenase (IDO)/tryptophan 2,3-dioxygenase (TDO) represents one of the most studied pathways for immunosuppression in tumor tissues. However, the pro-tumor effects induced by Trp metabolism remain controversial.

**Methods:**

The paraffin sections of tumor tissues were obtained from patients with liver cancer and examined by immunohistochemical staining to investigate the role of Trp metabolic enzymes. To further confirm the pro-tumor effects induced by TDO2, we established TDO2 overexpression SMC-7721 and HepG2 liver cancer cell lines, and western blotting, cell proliferation, and colony formation were evaluated. Meanwhile, liver cancer subcutaneous mice models were established, and the tumorigenic rates of SMC-7721 cells, tumor volume and survival of bearing mice were calculated. In addition, the survival data of liver cancer patients from The Cancer Genome Atlas (TCGA) database were downloaded to analyze the effect of TDO2 expression on the survival of patients with liver cancer.

**Results:**

Here, we showed that constitutive TDO2 expression gave rise to liver cancer through upregulation of Trp metabolism. And the TDO2 expression was positively correlated with the poor prognosis in liver cancer patients. TDO2 expression in tumor cells accounted for the release of kynurenine (Kyn), which activated aryl hydrocarbon receptor (AhR) to promote liver cancer cells proliferation. Mechanistically, we found that AhR expression contributed to the secretion of Interleukin-6 (IL-6), thereby promoting tumor cells proliferation through the STAT3 and NF-kB/TIM4 signals. Interrupt of AhR signals by PDM2 revealed improved outcomes in subcutaneous tumor-bearing mice.

**Conclusions:**

Together, our study showed that the TDO2/Kyn/AhR/IL-6 signaling pathway was a novel mechanism underlying the malignancy of liver cancer, and suggested that AhR signals might be a valuable therapeutic target for tumor therapy.

## Background

Liver cancer is the fifth most common form of cancer, with a third cause of cancer associated death worldwide [1]. Liver cancer is an aggressive disease with poor overall survival [2]. Only 20 % of liver cancer patients are still alive one year after diagnosis in the United Kingdom [3]. Despite the high incidence of liver cancer, there is a startling lack of therapeutic interventions. Of all diagnosed liver cancer patients, most patients take a cure of surgery combining with chemotherapy or immunotherapy. And most patients have a remaining lifespan of 4–6 months due to the advance of disease [4]. Therefore, there is an urgent demand to explore the underlying mechanism of tumor progression and discover innovative targets to eliminate tumor cells.

Tumor development is regulated by a diverse of factors, including mutations of anti-/oncogenes, tumor microenvironment, cancer stem cells, and abnormal tumor metabolic pathways. Notably, upregulation of Trp metabolism induced by IDO/TDO enzymes represents one of the most important pathways for tumor occurrence and development [5]. IDO1 and TDO2 enzymes catalyze Trp oxidation to Kyn, which are frequently observed in tumor tissues and tightly correlated with the poor prognosis in patients [6, 7]. Kyn synthesis has been demonstrated to promote tumorigenicity and growth by activating pro-survival signaling pathways, including PI3K/AKT and Wnt/β-catenin signals [8]. Compelling studies have suggested that IDO1 expression in tumor cells was linked to sustained tumor growth and the presence of immunosuppression [9]. Meanwhile, upregulation of IDO1 was observed in a series of tumor types in response to the infiltration reaction [10]. Despite the pro-tumor effects of IDO1, the specific role of TDO2 in cancer remains controversial.

IL-6 is a member of the IL-6 family, consisting of polypeptide cytokines and acting as both pro-inflammatory and anti-inflammatory molecules. Enhanced expression of IL-6 was observed in most human liver cancer tissues, in which it was produced in an autocrine manner in tumor cells [11]. Increasing evidence suggested that IL-6 plays an important role to drive tumor progression in several tumor types [12]. Mechanistically, IL-6 mediated the activation of pro-survival signals, especially the JAK/STAT3 signaling pathway, to facilitate tumor growth and distant metastasis [13]. Recent studies have implicated that cytosolic transcription factor AhR is involved in Kyn associated tumor progression, and could transcriptionally induce the expression of IL-6 [14]. However, the potential role of IL-6 in tumor-associated Trp metabolism still remains incompletely understood.

In our study, we now demonstrated that elevated expression TDO2 mediated liver cancer progression through upregulation of Trp metabolism. Constitutive TDO2 expression gave rise to the metabolic production of Kyn, resulting in the activation of AhR. In mechanism, we found that activation of AhR contributed to the upregulation of IL-6, which further promotes liver cancer development through a STAT3 and NF-kB/TIM4 dependent manner. Inhibition of AhR signals exhibited improved tumor suppressive effects, which described novel sight for liver cancer therapy.

## Methods

### Cell line and reagents

Human liver cancer cell lines SMC-7721 and HepG2 cells were obtained from the Key Laboratory of Hubei Province for Digestive System Disease (China) and cultured in RMPI-1640 complete culture medium (Gibco, USA) containing 10 % fetal bovine serum (Gibco, USA) at 37 °C in a humidified atmosphere of 5 % CO_2_. Doxorubicin (Dox), Kyn, human recombinant IL-6, and 5-Fluorouracil (5-FU) were purchased from Sigma (USA). AhR inhibitor PDM2 and NF-kB inhibitor JSH-23 were purchased from Selleck (USA).

### Tumor tissues collection from patients

The paraffin sections of tumor tissues were obtained from Fujian Medical University Union Hospital. Samples were divided into two groups, high stage (H-S, stage T2–T4) and low stage (L-S, stage T1a, and T1b) according to the pathological diagnosis. All patients agreed to participate in the study and were informed with written consent. The clinical experiments were performed according to the Declaration of Helsinki. This study was approved by the Ethics Committee of the Fujian Medical University Union Hospital.

### Cell proliferation

Tumor cells (3000 cells per well) treated in advance were seeded in a 96-well plate and incubated with 10 µl per well Cell Counting Kit-8 (CCK-8, Solarbio, China) reagent after 24, 48, and 72 h, respectively. Cell proliferation rates were detected using a microplate reader (BD, USA) at 450 nm.

### Colony formation

SMC-7721 and HepG2 cells were seeded in six-well plates (500 cells per well) and cultured with RMPI-1640 complete culture medium at 37 °C for 10 days. Colonies were fixed with methyl alcohol and stained with 4 % crystal violet solution. The colony numbers in each group were washed before counting.

### Western blotting

Tumor cells were collected and lysed in RIPA buffer (Byotime, China) with protease and phosphatase Inhibitor Cocktails (Biyuntian, China). Aliquots of 10 µg protein samples were separated by electrophoresis on 10 % SDS-polyacrylamide gels and transferred to a methanol‐pretreated PVDF membrane (Thermo, USA). Then samples were blocked with 5 % BSA in TBST and incubated with anti‐TDO2 (1:1000, Abcam, UK), anti-IDO1 (1:1000, Abcam, UK), anti‐AhR (1:1000, Abcam, UK), anti-STAT3 (1:1000, Abcam, UK), anti-TIM4 (1:1000, Abcam, UK) and anti‐β‐actin (1:1000, Abcam, UK) primary antibodies. Samples were visualized with an HRP‐conjugated secondary antibody (1:1000, Thermo, USA) and western blotting analysis system (Tanon, China).

### Transfection and plasmids

For TDO2 overexpression, cDNAs for human TDO2 were obtained from Ruibo Inc, China, The TDO2 cDNAs were inserted into pLVX-EF1α-IRES-Puro lentiviral vector (Takara, Japan) for stable overexpression in SMC-7721 and HepG2 cells. The overexpression of TDO2 was examined by western blotting.

### Immunohistochemical staining

Paraffin-embedded tumor tissue sections were cut. After dewaxing and rehydration, sections were treated with antigen retrieval solution for 15 min at 100 °C. Subsequently, samples were blocked with 5 % BSA in PBST for 30 min. Then slides were incubated with anti-TDO2 (1:200, Abcam, UK), anti-IDO1 (1:200, Abcam, UK), anti-IL-6 (1:200, Abcam, UK), anti-Kyn (1:200, Abcam, UK), anti-pSTAT3 (1:200, Abcam, UK), anti-NF-kB (1:200, Abcam, UK) antibodies overnight at 4 °C. After that, samples were incubated with HRP-conjugated secondary antibody (1:1000, Thermo, USA), developed by 3,3-diaminobenzidine solution and counterstained with hematoxylin. The intensity of protein expression in immunohistochemistry was determined by Image-Pro Plus 6.0 software. 8 tumor tissues from 8 patients were included in each group. 10 fields in each tumor tissue were analyzed and the mean of 10 values was determined as the expression intensity of this tumor tissues.

### Animal protocols

Female 6–8-week-old NOD-SCID mice were obtained from Huafukang (Beijing, China). Mice were bred in-house and fed on a standard animal chow diet, and raised in the SPF room. The animal protocol of this study was approved by the Institutional Animal Care and Use Committee of Fujian Medical University Union Hospital. The animal studies were conducted following the Public Health Service Policy and complied with the WHO guidelines for the humane use and care of animals.

For tumorigenic analysis, 1 × 10^5^ SMC-7721 cells resuspended in 50 µl PBS were injected subcutaneously (n = 10 in each group), and the tumorigenic rates were calculated on day 20. For tumor volume and survival analysis, 10^6^ SMC-7721 cells were injected into NOD-SCID subcutaneously (n = 6 in each group). When the tumor size reached 600 mm^3^, mice were treated with PBS, DOX (5 mg/kg), 5-FU (10 mg/kg), PDM2 (5 mg/kg), or combining treatment twice a week. Tumor volume and survival of mice were recorded every day (end point, day 80). The calculation formula of tumor volume is: tumor volume = length × width 2/2.

### Statistical analysis

The overall survival of liver cancer patients was downloaded from https://www.cbioportal.org/. All data were compared using either Student’s t-test, one-way or two-way analysis of variance with Tukey multiple comparison posttest in GraphPad Prism 8 (USA) to determine statistical significance between different groups. The survival was analyzed by Kaplan–Meier survival analysis. *p < 0.05; **p < 0.01; ns, no significant difference. Each experiment was performed three times independently.

## Results

### TDO2 expression correlated with liver cancer development

**Fig. 1:**
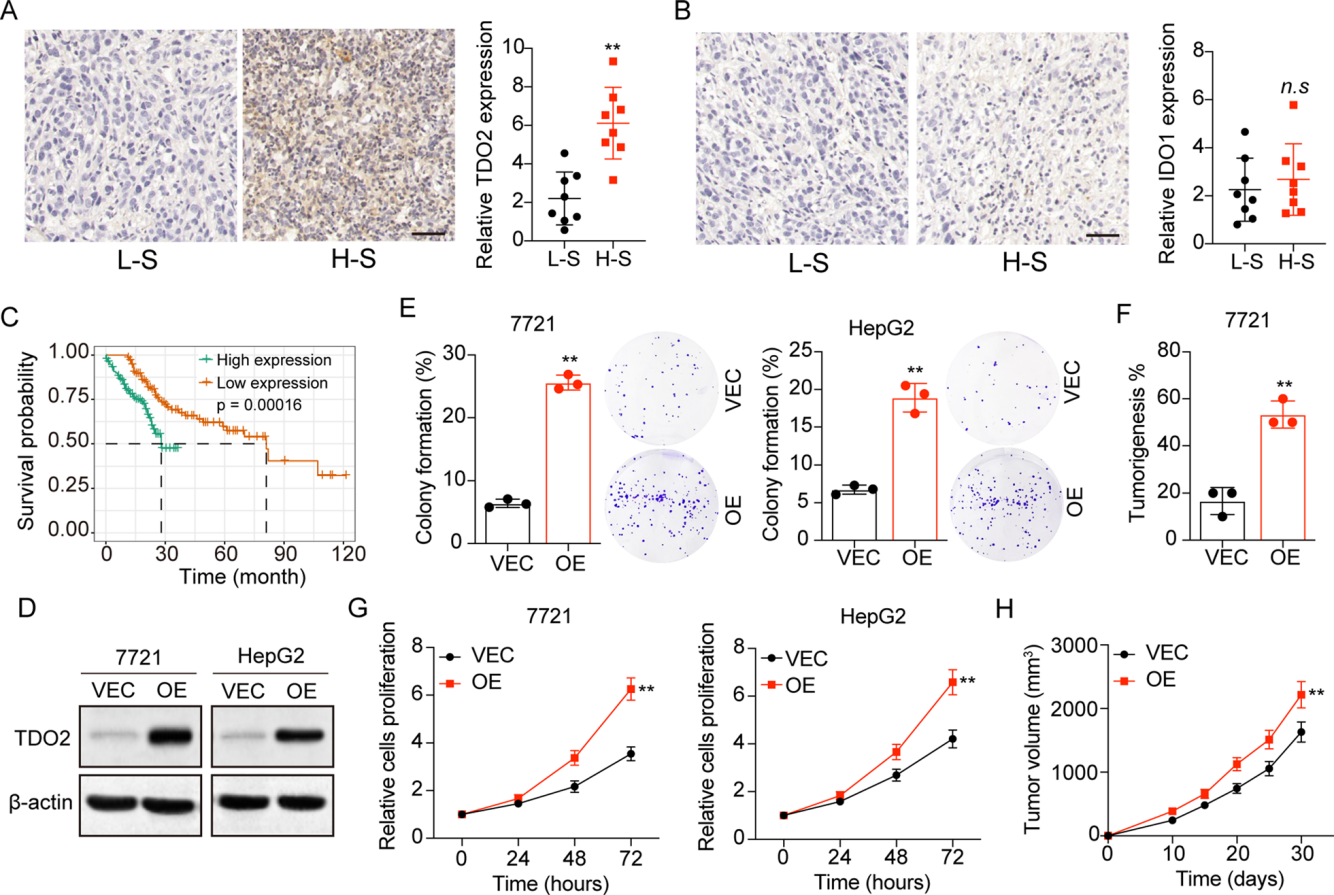
TDO2 expression correlated with liver cancer development. **A** Immunohistochemical staining of TDO2 in high stage (H-S) and low stage (L-S) tumor tissues from liver cancer patients. The intensity of TDO2 expression was calculated in each group (n = 8). The scale bar is 100 μm. **B** Immunohistochemical staining of IDO1 in high stage (H-S) and low stage (L-S) tumor tissues from liver cancer patients. The intensity of IDO1 expression was calculated in each group (n = 8). The scale bar is 100 μm. **C** The survival analysis of liver cancer patients divided into high TDO2 expression (n = 134) and low TDO2 expression (n = 136) groups using TCGA database. **D** Western blotting of TDO2 in SMC-7721/HepG2 and TDO2 overexpression SMC-7721/HepG2 cells. **E** The colony formation rates of SMC-7721/HepG2 (VEC) and TDO2 overexpression SMC-7721/HepG2 (OE) cells. **F** The cell proliferation of SMC-7721/HepG2 (VEC) and TDO2 overexpression SMC-7721/HepG2 (OE) cells using CCK-8 analysis. **G** The tumorigenic capability of SMC-7721 (VEC) and TDO2 overexpression SMC-7721 (OE) cells in immunodeficient mice. **H** The tumor volume of SMC-7721 (VEC) and TDO2 overexpression SMC-7721 (OE) bearing mice. *p < 0.05, **p < 0.01, n.s, no significant difference

The expression of IDO and TDO, the rate-limiting enzymes in the Trp/Kyn metabolism pathway, was observed in various cancer, including hepatic carcinoma [6, 7, 15, 16]. To investigate the role of Trp metabolic enzymes, we examined the expression of IDO1 and TDO2 in tumor tissues from liver cancer patients. Tumor tissues were divided into two groups, high stage (H-S, stage T2–T4) and low stage (L-S, stage T1a, and T1b). Tumor tissues in the H-S group exhibited enhanced expression of TDO2 (Fig. 1A), and no significant difference was observed in IDO1 expression (Fig. 1B). Importantly, poor overall survival of liver cancer patients with high TDO2 expression was validated in TCGA database (Fig. 1C). These results indicated that TDO2 was responsible for the poor prognosis in liver cancer patients. To further confirm the pro-tumor effects induced by TDO2, we established TDO2 overexpression SMC-7721 and HepG2 liver cancer cell lines (Fig. 1D), and cell proliferation or colony formation were evaluated. In SMC-7721 and HepG2 cells, overexpression of TDO2 significantly strengthened the capability of colony formation (Fig. 1E) in tumor cells. Meanwhile, a higher tumorigenic rate of TDO2 overexpression SMC-7721 cells was found in immunodeficient mice (Fig. 1F), and TDO2 overexpression SMC-7721 revealed strengthened cell proliferation capability (Fig. 1G) and rapid tumor growth in vivo (Fig. 1H), when compared to the SMC-7721 vector group. Collectively, those findings supported the notion that TDO2 promoted liver cancer progression.

### TDO2 modulated Trp/Kyn metabolism to regulate tumor progression

**Fig. 2:**
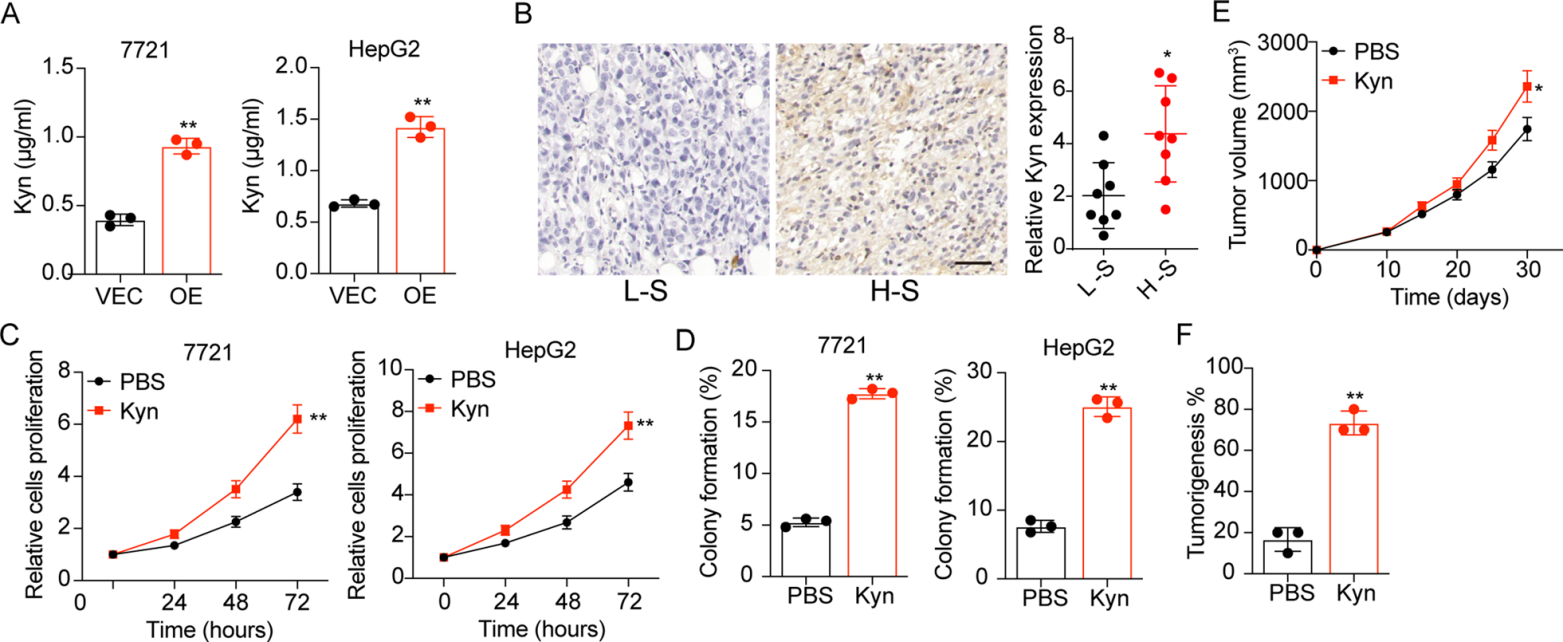
TDO2 modulated Trp/Kyn metabolism to regulate tumor progression. **A** 10^5^ SMC-7721/HepG2 (VEC) and TDO2 overexpression SMC-7721/HepG2 (OE) cells were cultured in 2 ml culture medium on day 0. After 48 h of culture, the Kyn concentration in the supernatant was determined using Elisa analysis. **B** Immunohistochemical staining of Kyn in high stage (H-S) and low stage (L-S) tumor tissues from liver cancer patients. The scale bar is 100 μm. **C** Cell proliferation of SMC-7721/HepG2 treated with PBS or Kyn (10 µM). **D** The colony formation rates of SMC-7721/HepG2 treated with PBS or Kyn (10 µM). **E** The tumor volume of SMC-7721 pre-treated with PBS or Kyn (10 µM, 72 h). **F** The tumorigenic capability of SMC-7721 pre-treated with PBS or Kyn (10 µM, 72 h). *p < 0.05, **p < 0.01, n.s, no significant difference

IDO/TDO induced Kyn production is a key pathway facilitating tumor progression. Indeed, enhanced secretion of Kyn was detected in the supernatant of TDO2 overexpression SMC-7721 and HepG2 cells using Elisa analysis (Fig. 2A). And tumor tissues from patients with high TDO2 expression exhibited increased Kyn expression using immunohistochemistry (Fig. 2B), indicating that TDO2 modulated the Kyn production in tumor cells. Subsequently, we further investigated the influence of Kyn on liver cancer cells proliferation, and Kyn was added into the culture medium of SMC-7721 and HepG2 cells. Consequently, Kyn treatment strengthened the proliferation (Fig. 2C) and colony formation (Fig. 2D) of SMC-7721 and HepG2 cells. Similar results were found in vivo (Fig. 2E and F). Taken together, those results suggested that TDO2 promoted Kyn production to regulate liver cancer progression.

### Trp metabolite Kyn promoted IL-6 secretion through AhR

**Fig. 3:**
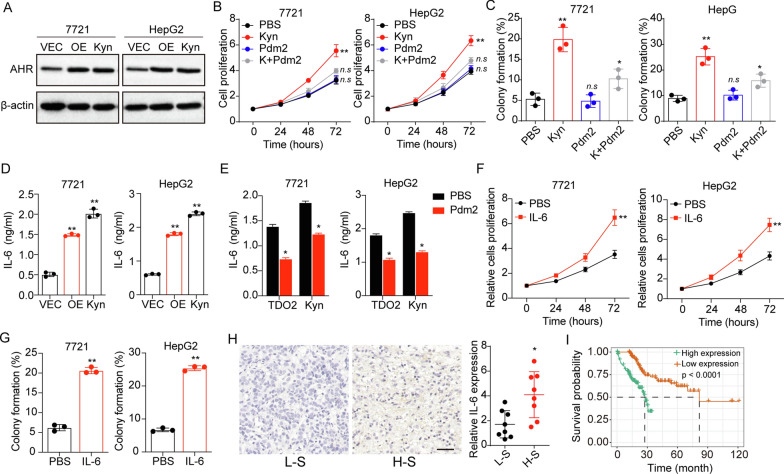
Trp metabolite Kyn promoted IL-6 secretion through AhR. **A** Western blotting of AhR in SMC-7721/HepG2 (VEC), TDO2 overexpression SMC-7721/HepG2 (OE) and SMC-7721/HepG2 treated with Kyn (10 µM) (Kyn). **B** SMC-7721/HepG2 cells were cultured with medium containing Kyn (10 µM) or not. Then cells were treated with PBS or Pdm2 (10 nM) and the cell proliferation was detected. **C** The colony formation of SMC-7721/HepG2 cells in (**B**). **D** 10^5^ SMC-7721/HepG2 (VEC), TDO2 overexpression SMC-7721/HepG2 (OE) and Kyn (10 µM) treated SMC-7721/HepG2 (Kyn) cells were cultured in 2 ml culture medium. After 48 h, the IL-6 concentration in the supernatant was determined using Elisa analysis. **E** 10^5^ TDO2 overexpression SMC-7721/HepG2 and Kyn (10 µM) treated SMC-7721/HepG2 cells were cultured in 2 ml culture medium. PBS or PDM2 (10 nM) was added into the culture medium. After 48 h, the IL-6 concentration in the supernatant was determined using Elisa analysis. **F** Cell proliferation of SMC-7721/HepG2 cells treated with PBS or IL-6 (10ng/ml). **G** Colony formation of tumor cells in (**F**). **H** Immunohistochemical staining of IL-6 in high stage (H-S) and low stage (L-S) tumor tissues from liver cancer patients. The scale bar is 100 μm. **I** The survival analysis of liver cancer patients divided into high IL-6 expression (n = 134) and low IL-6 expression (n = 136) groups using TCGA database. *p < 0.05, **p < 0.01, n.s, no significant difference

Previous studies have demonstrated that Kyn could activate the AhR signaling pathway to modulate tumor progression [14]. Thus, we sought to validate the hypothesis that Kyn promoted liver cancer development through an AhR dependent manner. As anticipated, TDO2 overexpression and Kyn treatment upregulated the expression of AhR in SMC-7721 and HepG2 cells (Fig. 3A). To explore the contribution of AhR activation to tumor progression, we used PDM2, an AhR inhibitor, to treated liver cancer cells cultured with Kyn. As shown in Fig. 3B, inhibition of AhR suppressed the cell proliferation induced by Kyn, as well as the colony formation (Fig. 3C), indicating that Kyn mediated AhR activation to promote liver cancer development. IL-6 is transcriptionally induced via the AhR, which was involved in the progenitor/stem cells proliferation in liver cancer. In fact, we found elevated IL-6 secretion in the supernatant of TDO2 overexpression or Kyn treated SMC-7721 and HepG2 cells (Fig. 3D), and suppression of AhR reduced IL-6 secretion induced by TDO2 or Kyn (Fig. 3E). Next, we wondered whether autocrine IL-6 could stimulate cell proliferation of liver cancer. Indeed, treatment of human recombinant IL-6 remarkably strengthened the proliferation (Fig. 3F) and colony formation (Fig. 3G) of SMC-7721 and HepG2. In consistent, elevated expression of IL-6 was observed in H-S tumor tissues (Fig. 3H), and poor overall survival was found in IL-6 high expression patients with liver cancer (Fig. 3I). Together, those results implicated that Kyn promoted IL-6 expression through AhR to promote liver cancer progression.

### STAT3 and TIM4/NF-kB were involved in the IL-6 induced tumor progression

**Fig. 4:**
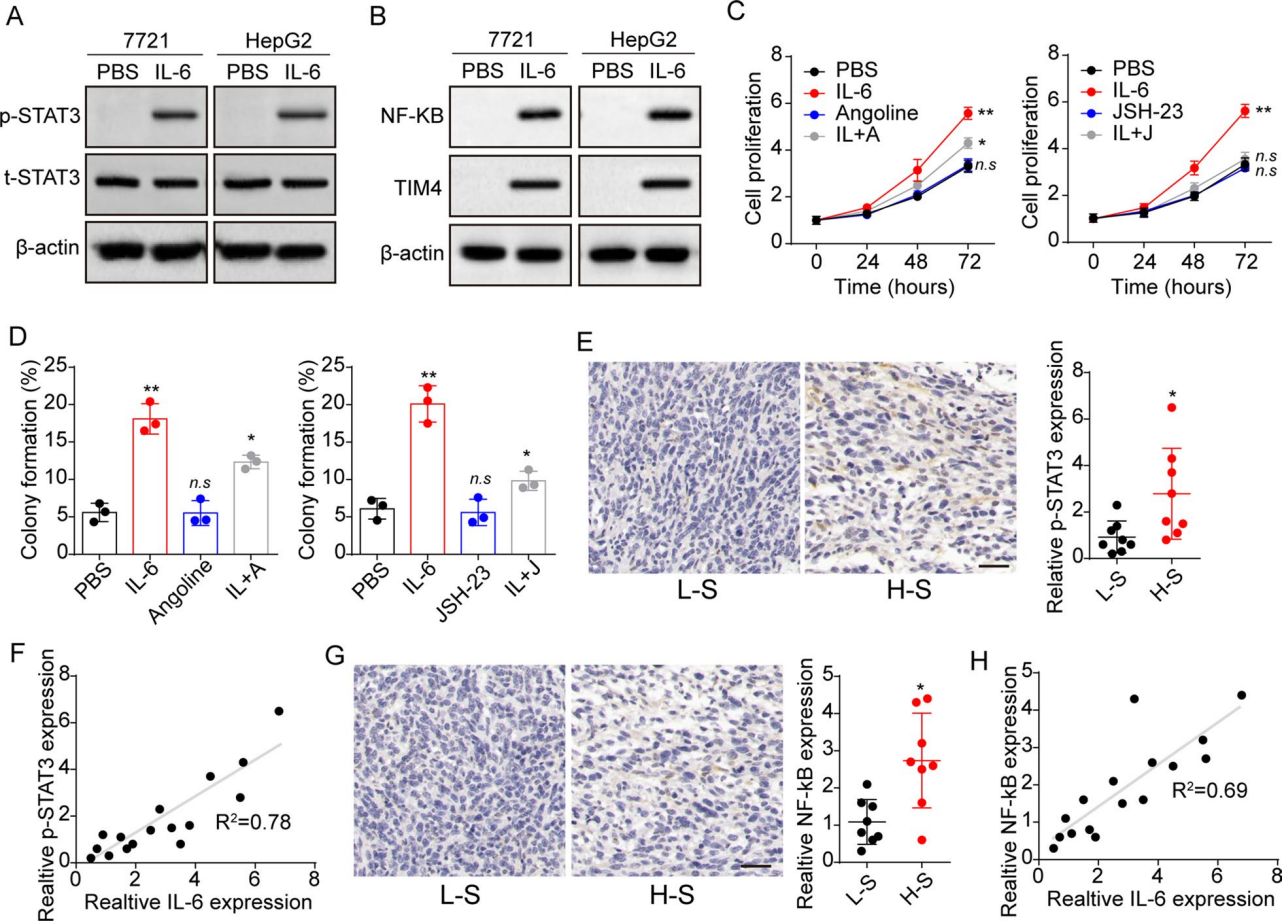
STAT3 and TIM4/NF-kB were involved in the IL-6 induced tumor progression. **A** Western blotting of phosphorylated STAT3 and STAT3 in SMC-7721 and HepG2 cells treated with PBS or IL-6 (10 ng/ml). **B** Western blotting of NF-kB and TIM4 in SMC-7721 and HepG2 cells treated with PBS or IL-6 (10 ng/ml). **C** SMC-7721/HepG2 cells were cultured with medium containing IL-6 (10 ng/ml) or not. Then cells were treated with PBS, angoline (20 nM) or JSH-23 (5 nM) and the cell proliferation was detected. **D** Colony formation rates of SMC-7721 cells in (**C**). **E** Immunohistochemical staining of phosphorylated STAT3 in high stage (H-S) and low stage (L-S) tumor tissues from liver cancer patients. The scale bar is 100 μm. F, correlation analysis between IL-6 and p-STAT6 in tumor tissues from liver patients using immunohistochemistry (n = 16, R^2^ = 0.78). **G** Immunohistochemical staining of NF-kB in high stage (H-S) and low stage (L-S) tumor tissues from liver cancer patients. The scale bar is 100 μm. **H** correlation analysis between IL-6 and NF-kB in tumor tissues from liver patients using immunohistochemistry (n = 16, R^2^ = 0.78). *p < 0.05, **p < 0.01, n.s, no significant difference

IL-6 activated STAT3 and NF-kB/TIM4 signals to regulate tumor progression. We therefore analyzed STAT3 and NF-kB/TIM4 expression in SMC-7721 and HepG2 cells. Elevated expression of phosphorylated STAT3 (Fig. 4A) and NF-kB/TIM4 (Fig. 4B) was observed in tumor cells after IL-6 stimulation. To further confirm the role of STAT3 and NF-kB/TIM4, we used STAT3 inhibitor angoline and NF-kB inhibitor JSH-23 to treat SMC-7721 and HepG2 cells. Consistently, suppression of STAT3 and NF-kB reduced the proliferation (Fig. 4C) and colony formation (Fig. 4D) of IL-6 treated tumor cells. More importantly, we determined the expression of phosphorylated STAT3 and NF-kB in tumor tissues from patients, and an additional increase of p-STAT3 (Fig. 4E) was found in H-S tumor tissues. Additionally, the expression analysis revealed a correlation between IL-6 and p-STAT3 expression in tumor tissues (Fig. 4F). The similar results were observed in NF-kB expression (Fig. 4G and H). Those results suggested that STAT3 and TIM4/NF-kB signals were involved in the IL-6 associated liver cancer development.

### Suppression of AhR signals improved the anticancer effects of chemotherapy

**Fig. 5:**
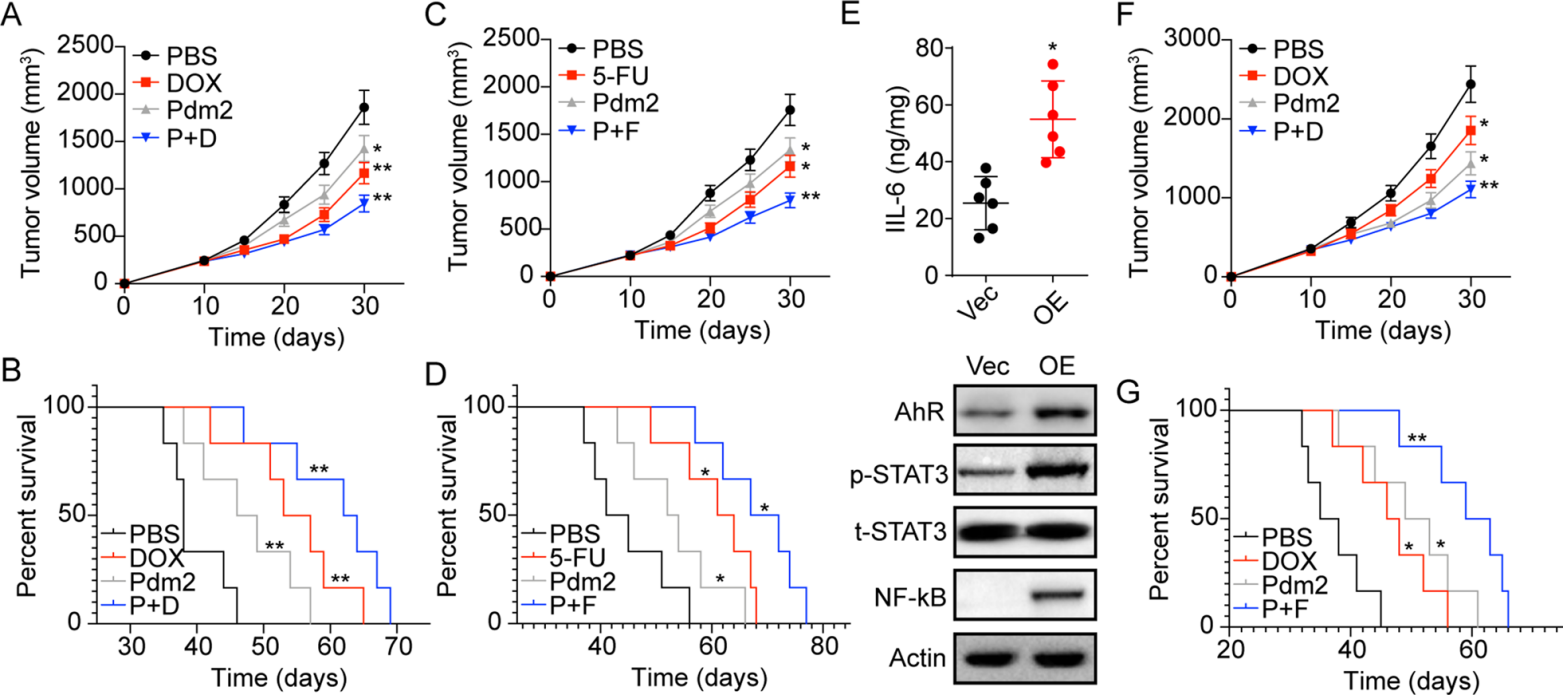
Suppression of AhR signals improved the anticancer effects of chemotherapy. **A** The tumor volume of SMC-7721 bearing mice treated with PBS, PDM2, DOX and PDM2 combining DOX. **B** Survival analysis of SMC-7721 bearing mice treated with PBS, PDM2, DOX and PDM2 combining DOX. **C** The tumor volume of SMC-7721 bearing mice treated with PBS, PDM2, 5-FU and PDM2 combining 5-FU. **D** Survival analysis of SMC-7721 bearing mice treated with PBS, PDM2, 5-FU and PDM2 combining 5-FU. E, Expression of IL-6, AhR, p-STAT3, total STAT3 and NF-kB in Vector (Vec) or TDO2 overexpression (OE) SMC-7721 tumor tissues using Elisa or western blotting analysis. **F** The tumor volume of TDO2 overexpression SMC-7721 bearing mice treated with PBS, PDM2, DOX and PDM2 combining DOX. G, Survival analysis of TDO2 overexpression SMC-7721 bearing mice treated with PBS, PDM2, DOX and PDM2 combining DOX. *p < 0.05, **p < 0.01

As TDO2 expression contributed to liver cancer progression through Kyn/AhR/IL-6 signaling, we next examined the effects of combination therapy using AhR inhibitor PDM2 together with chemotherapeutic DOX or 5-FU. In NOD-SCID mice with SMC-7721 tumors, both chemotherapeutic DOX and AhR inhibitor PDM2 resulted in tumor suppression and prolonged overall survival. Notably, the combination group revealed improved anticancer effects compared with the monotherapy group (Fig. 5A and B). Similar results were observed in PDM2 and 5-FU combination (Fig. 5C and D). Subsequently, we evaluated whether PDM2 treatment possessed improved tumor suppressive effects in TDO2 overexpression tumors. Consistently, activation of AhR/IL-6/STAT3/ NF-kB signaling was observed in TDO2 overexpression tumor tissues (Fig. 5E). Subsequently, mice with TDO2 overexpression SMC-7721 tumors were treated with PBS, DOX, PDM2, and DOX combining PDM2. Of note, limited tumor suppressive effects were found in DOX treated tumor-bearing mice. The phenomenon might be caused by the activation of STAT3 and NF-kB signals, which have been reported to mediate drug resistance in several tumor types. However, PDM2 obviously strengthened the anticancer effects of DOX (Fig. 5F and G), indicating that suppression of AhR could efficiently improve the outcome of chemotherapy, describing a novel strategy for liver cancer treatment.

## Discussion

Constitutive IDO/TDO expression is a key mechanism to promote tumor sustained growth and immunosuppression in patients, which represents an ideal target for tumor therapy [9, 17, 18]. Previous clinical studies have suggested the IDO1 inhibitors, such as 1-methyl-tryptophan, exhibited remarkable tumor suppressive effects in patients with solid tumors [19, 20]. Despite the pro-tumor efficacy of IDO1 in tumor development and therapy, the role of TDO2, an IDO1 isozyme, remained poorly investigated. In this study, we identified a novel mechanism in which TDO2, through Trp/Kyn metabolism, activated AhR/IL-6/STAT3/NF-kB signaling and promoted liver cancer progression. Comparing tumor tissues from clinical liver cancer patients and TCGA database, we examined the TDO2 activity and the downstream metabolites, that promoting liver cancer progression. Using liver cancer cell lines and subcutaneous mice models, we demonstrated that Kyn produced by TDO2/Trp metabolism activated AhR/IL-6 pathway in liver cancer, eventually resulting in upregulation of the NF-kB/TIM4/STAT3 signals and cancer development. These findings described novel mechanistic sight and strengthened the concept for Trp metabolism associated with tumor progression.

The Trp metabolite Kyn has been defined as AhR ligands in several tumor types and immune cells [14, 21]. Notably, Liu and her colleagues reported that Kyn derived from tumor repopulating cells mediated the activation of AhR in T cells to suppress the inflammatory response in the tumor microenvironment [22]. And the Kyn produced by Trp catabolizing enzyme IDO in tumor cells has been demonstrated to facilitate AhR activation and tumor growth in an immune independent manner [14, 23]. Our study further provided evidence to suggest the TDO2 was equally capable of producing Kyn to mediate the AhR pathway activation in liver cancer cells.

To our knowledge, this is the first study to define a signaling pathway by which TDO2 facilitated cancer cell proliferation via an autocrine IL-6 manner. Compelling reports have demonstrated that IDO promoted the IL-6 expression in tumor cells [24–26], and in turn, IL-6 was reported to upregulate IDO expression through a JAK/STAT signaling pathway [27, 28]. Notably, the expression of AhR has been shown to mediate the IL-6 expression in ovarian cancer [29], and Kyn derived from Trp metabolism could efficiently promote the activation of AhR signals in a diverse of tumor cells [21]. Our study further identified the correlation between those findings, in which we demonstrated that TDO2 mediated the IL-6 production through Trp/Kyn/AhR signaling.

The expression of IL-6 has been demonstrated to correlate with the tumor development in several tumor types [12, 30, 31]. Mostly, autocrine or paracrine IL-6 regulated tumor progression and promoted the conversion of non-stem cancer cells into cancer stem-like cells through JAK/STAT3 signaling pathway [31–33]. However, increasing evidence suggested that T-cell immunoglobulin domain and mucin domain 4 (TIM4) and NF-kB are involved in the tumor progression induced by IL-6 in non-small cell lung cancer [23]. Here, our study showed that constitutive STAT3 activation was controlled by autocrine IL-6. Notably, upregulation of TIM4 and NF-kB was observed in IL-6 treated liver cancer cells and tumor tissues from patients. It was likely that both STAT3 and NF-kB signals were required for IL-6 induced liver cancer progression, though the specific underlying mechanism remains unclear. However, blockade of AhR directly suppressed the IL-6 secretion and tumor development induced by TDO2. More importantly, the combination of AhR inhibitor PDM2 and chemotherapy revealed strengthened tumor suppressive effects in subcutaneous tumor-bearing mice, which descried an innovative approach for clinical tumor therapy targeting Trp metabolism.

## Conclusions

In conclusion, our study described a novel mechanism, in which TDO2 mediated the Trp metabolism and activation of the Kyn/AhR/IL-6 signaling pathway in tumor cells. Those findings demonstrated the crucial roles of TDO2 activity in driving tumorigenicity and development in liver cancer. And suppression of AhR signals revealed improved anticancer effects, providing insights for further clinical trials that exploits a combination of AhR inhibitors and chemotherapy.

## Data Availability

The datasets used and/or analyzed during the current study are available from the corresponding author on reasonable request.

## Abbreviations

Trp: Tryptophan
IDO: Indoleamine 2,3-dioxygenase
TDO: Tryptophan 2,3-dioxygenase
Kyn: Kynurenine
AhR: Activated aryl hydrocarbon receptor
IL-6: Interleukin-6
TIM4: T-cell immunoglobulin domain and mucin domain 4
